# Microphysiological systems for metastasis research: a stepwise approach

**DOI:** 10.1007/s13402-025-01110-4

**Published:** 2025-10-22

**Authors:** Vira Sharko, Ignacio Ochoa, Estela Solanas

**Affiliations:** 1 Tissue Microenvironment (TME) Lab, Aragon Institute of Engineering Research (I3A), University of Zaragoza, Zaragoza, 50018 Spain; 2 Aragon Institute for Health Research (IIS Aragón), Zaragoza, 50018 Spain; 3 Center for Biomedical Research in the Network in Bioengineering, Biomaterials and Nanomedicine (CIBER-BBN), Zaragoza, 50018 Spain

**Keywords:** Microphysiological systems, Microfluidics, Cancer, Metastasis, Tumor microenvironment

## Abstract

Metastasis, the leading cause of cancer-related mortality, is a complex process involving tumor cell detachment from the primary site, survival and dissemination through the circulation, and colonization of distant organs. At each stage, tumor cells face adaptive pressures from successive biological and biomechanical challenges in the local microenvironment, which collectively shape their progression. Traditional in vitro models often fail to replicate these dynamics, while animal models are limited by species differences and restricted real-time monitoring. Microphysiological systems (MPS) have emerged as powerful tools to address these limitations, delivering physiologically relevant cues and precise experimental control to recapitulate step-specific metastatic contexts. This review outlines recent advances in MPS designs for modeling critical hallmarks of metastasis, beginning with matrix interactions, stromal cells, and mechanical forces from the tumor microenvironment that drive epithelial-mesenchymal transition and invasion. The discussion then transitions to MPS that reproduce vascular physiology during intravasation, circulation, and extravasation, and concludes with organ-specific environments for studying colonization and organotropic behavior in the final stages of metastasis. Additionally, common MPS configurations, categorized into horizontal and vertical compartmental arrangements, and strategies for integrating vascularization are explored. Together, these advances highlight the potential of MPS in elucidating metastatic mechanisms and advancing targeted therapies.

## Introduction

Metastasis, the process by which tumor cells spread throughout the body, forming secondary tumors and causing organ dysfunction, stands as the leading cause of cancer-related mortality worldwide [1]. This journey involves tumor cell detachment from their primary site, infiltration into the bloodstream or lymphatics, survival amidst the challenges of circulation, extravasation into a secondary organ, and adaptation to the microenvironment of the distant site, all while evading immune surveillance and retaining their phenotypic plasticity and self-renewal capacities [2, 3]. This process, governed by the tumor-intrinsic malignancy and its interplay with the surrounding tumor microenvironment (TME), is highly complex and dynamic [4, 5].

The TME comprises stromal cells, immune cells, and blood vessels, interwoven with non-cellular elements like the extracellular matrix (ECM). This network is further shaped by chemical factors like oxygen and nutrient gradients, and mechanical cues like interstitial fluid flow and shear stress [6]. While initially acting as a tumor suppressor, the TME can adapt to promote metastasis in advanced cancer stages [4]. Recent research highlights specific TME elements as critical drivers of metastasis, triggering and modulating epithelial-mesenchymal transition (EMT) in tumor cells [7–9]. EMT enables tumor cells to detach from the primary tumor, navigate through the ECM, and progress through subsequent metastatic stages. It is also believed to facilitate cell entry into the vasculature and sustain a stem cell-like phenotype, while its reversal after extravasation — the mesenchymal-epithelial transition — is critical for organ colonization [7, 10, 11].

**Fig. 1:**
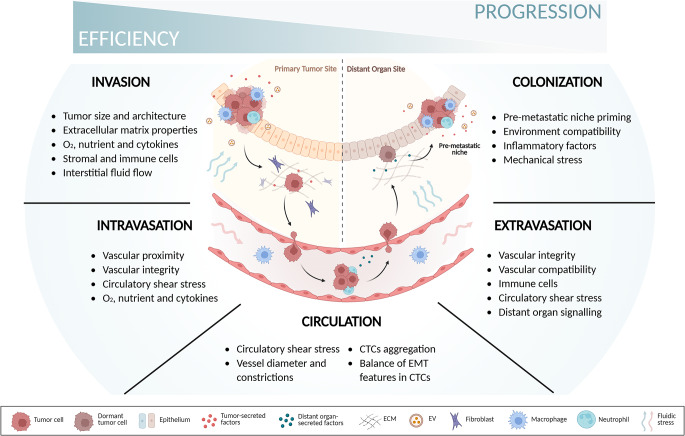
Schematic representation of metastatic progression in terms of efficiency, illustrating the key microenvironmental factors that influence tumor cell survival at each stage — from primary tumor detachment and invasion to intravasation into the blood vessel, circulation, extravasation, and ultimately colonization at a secondary site. This image was created using BioRender (https://biorender.com/). Abbreviations: CTCs, circulating tumor cells; EMT, epithelial-mesenchymal transition; ECM, extracellular matrix; EVs, extracellular vesicles

The initial phase of metastasis is highly efficient. Within its native microenvironment, the tumor thrives and remains protected, increasing its potential to spread as it grows [12, 13]. During this stage, tumor expansion impacts its surroundings both physically — by compressing nearby ECM and altering interstitial pressure — and biochemically — by elevating oxygen consumption, fiber deposition, and signaling to adjacent cells, among other effects (Fig. 1) [5, 14, 15]. As tumor cells approach the vasculature, tumor-secreted factors can induce vessel formation while disrupting the vascular barrier in nearby vessels, thereby increasing their chances of intravasating [16–18]. However, once in circulation, tumor cells face significant challenges from vascular architecture and fluidic forces, which hinder their survival and ability to adhere to the endothelium [14, 19]. Subsequently, extravasation and colonization remain highly unlikely events, occurring more frequently in organs receptive to tumor signaling, which promote vascular remodelling and microenvironmental preconditioning in distant organs, facilitating extravasation, metastatic growth, and eventual colonization [20–22]. The late stages of metastasis represent a major bottleneck in the progression of the disease, with only a small fraction of disseminated cells successfully establishing a secondary niche (Fig. 1) [23, 24].

Despite the overall inefficiency of metastasis, it remains a central focus of cancer research due to the significant treatment challenges it presents. These arise from the genetic heterogeneity and drug resistance acquired by tumor cells during metastatic progression [24, 25] compounded by the lack of reliable biomarkers and the limited sensitivity of current imaging techniques, often resulting in late diagnosis [26]. Overcoming these barriers requires advancements in detection methods, precision medicine, immunotherapy, and the development of robust preclinical models to facilitate the study of metastatic progression and the evaluation of treatment strategies [27].

The early stages of metastasis in the primary TME are typically investigated using conventional in vitro models, where tumor cells or aggregates are embedded in scaffolds and seeded onto culture plates or modified Boyden chambers [28]. While some of these systems incorporate ECM components and co-culture with TME cells, they often fail to reproduce key microenvironmental stimuli and offer limited tracking potential [29, 30]. In contrast, research on late-stage metastasis involving tumor-vascular interactions often relies on animal models, requiring the implantation of human tumor xenografts into mice to observe primary tumor progression or the injection of tumor cells to follow extravasation via intravital microscopy [19, 31]. Despite their physiological relevance, animal models are hindered by restricted monitoring capabilities and inherent genetic, immune, and metabolic differences from humans, which reduce their clinical relevance [32]. These limitations have driven an increased demand for alternative in vitro models that more accurately mimic and track tumor cell progression at each stage of metastasis, while also addressing the ethical and economic concerns associated with animal experimentation [33].

Recently, microfluidic technology has emerged as a promising solution to these challenges. Operating at micrometer scale, microphysiological systems (MPS) allow precise control over experimental conditions, enabling high-resolution, real-time monitoring of cellular dynamics throughout the metastatic process [32, 33]. Their versatile designs, particularly in organ-on-a-chip models, and their ability to deliver physiologically relevant biochemical and biophysical stimuli hold substantial potential for accurately modelling the influence of the cellular context during both tumor initiation [34, 35] and dissemination [20, 36]. In this context, MPS models metastasis, often viewed as a compartmentalized process in which tumor cells must overcome increasing adaptive pressures at each stage, have been engineered to reproduce the environmental cues specific to each step or transition. These models are able to capture the influence of the local ECM, mechanical cues and stromal cells during invasion step [8, 30] vascular physiology and fluidic stress during intravasation, circulation, and extravasation steps [18, 37, 38] and the target organ microenvironment during colonization step [39, 40]. This stepwise approach constitutes the basis of this review on MPS in metastasis research, offering an overview of the stimuli that govern tumor cell behavior at each step, the platform designs enabling these investigations, and the significant insights they have provided.

## Microphysiological systems for studying local invasion

Invasion is a pivotal step in metastasis, marking the point at which tumor cells acquire the necessary capabilities to survive and progress through the metastatic journey [3, 23]. This step is strongly influenced by the surrounding TME, which continuously evolves in response to oncogenic signaling, giving rise to changes in the ECM, molecular cues such as hypoxia and cytokines secreted by stromal and infiltrating immune cells, and physical forces driven by flow and pressure [4, 5]. Broadly, these elements can be classified into three categories, each exemplified by a key factor with particularly strong influence: the ECM as the dominant structural component, fibroblasts among cellular contributors, and interstitial fluid flow (IFF) as the principal mechanical stimulus.

In response to these cues, tumor cells activate EMT-related genes, amplifying pro-tumor signaling and reinforcing a feedback loop that sustains metastasis initiation [14, 24, 41]. During EMT, tumor cells undergo a series of molecular transformations that induce cytoskeletal rearrangements, shifting from intercellular adhesions to individual cell-ECM interactions. This transition leads to a more migratory phenotype, accompanied by the loss of cell polarity and cohesion at the tumor periphery [42–44]. In addition, increased secretion of proteases facilitates the breaching of the basement membrane (BM) in epithelial cancers and the degradation of the ECM, allowing the cells to migrate toward the vasculature [7, 41]. Understanding the influence of TME on tumor cell invasion is essential for developing effective strategies to prevent and treat early metastasis. To this end, MPS offer superior control over cellular and mechanical parameters compared to traditional compartmentalized systems like Boyden chambers, while also enabling real-time tracking and in-depth analysis of single-cell dynamics to capture the heterogeneity and phenotypic plasticity inherent in this stage of metastasis [29, 30].

### Modelling the influence of the extracellular matrix on invasion

The ECM, a complex network of macromolecules, provides structural, biochemical, and mechanical cues that regulate cell adhesion, behavior, and motility [42, 45, 46]. Its essential role in the initiation of metastasis is underscored by the fact that invasion involves the active displacement of tumor cells through a three-dimensional (3D) tissue environment, a process orchestrated by mechanical forces and cell-ECM interactions [43, 45–47]. Accordingly, invasion-focused MPS commonly incorporate ECM-mimicking scaffolds as a central design feature to accurately reproduce these dynamics in vitro. These matrices are typically confined within the central chamber of the device to leverage their ability to deliver chemoattractant gradients between two symmetrical side channels — one serving as the donor and the other as the recipient [35, 48–51]. ECM-mimicking materials, such as collagen I-based hydrogels and mixed-composition Matrigel [52, 53]support chemoattractant diffusion, allowing the formation and maintenance of stable gradients over time [54].

**Fig. 2:**
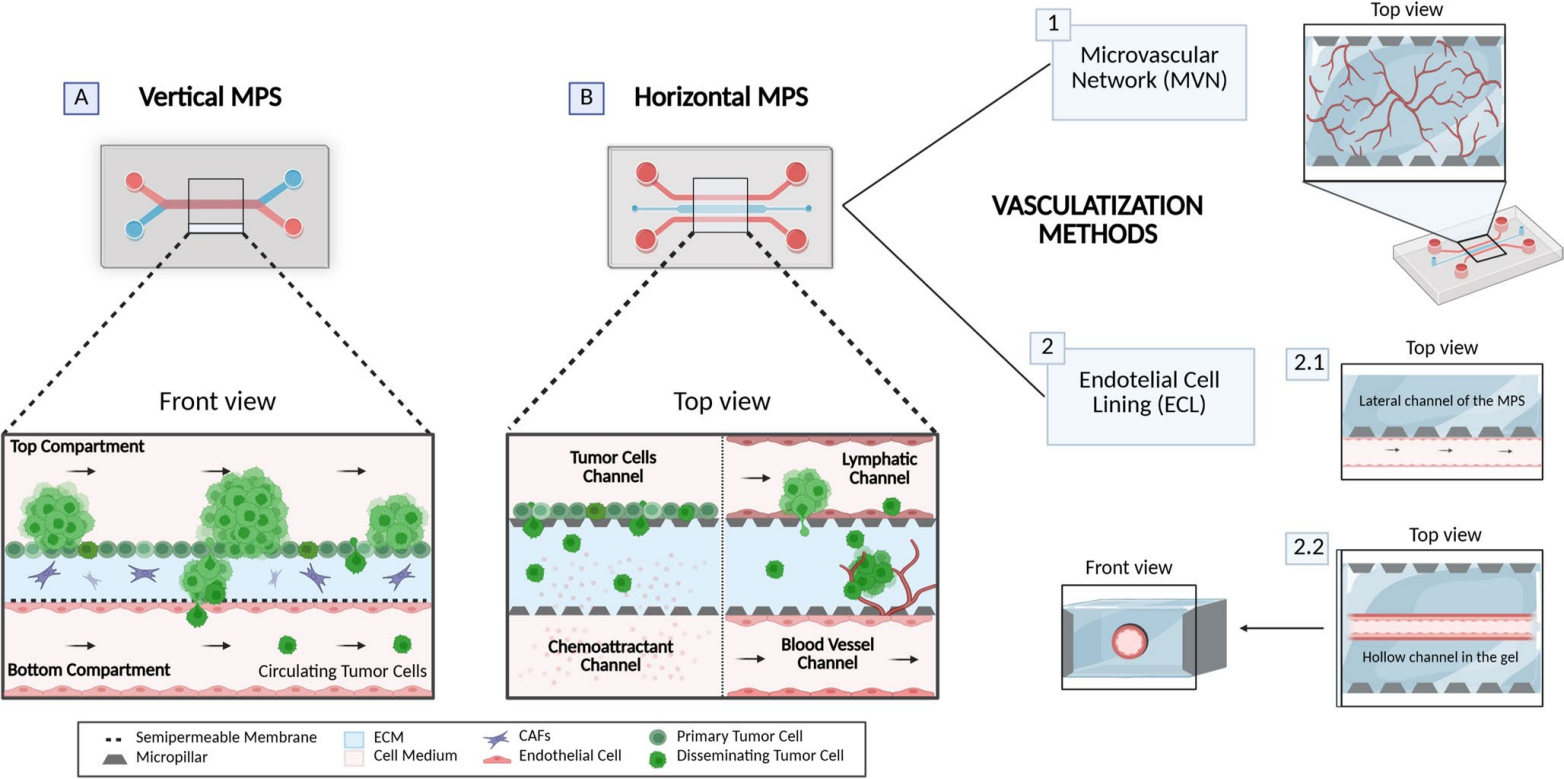
Schematic of the two main microphysiological systems (MPS) designs used to model metastasis: **A**) Vertical MPS and **B**) Horizontal MPS. The front and top views of the devices illustrate typical experimental setups used to simulate tumor cell invasion and intravasation based on described models [30, 50, 75]. The vascularization strategies for B) Horizontal MPS are shown on the right side of the image: (1) formation of a microvascular network (MVN) within the central matrix chamber, and (2) endothelial cell lining (ECL) either (2.1) along the gel wall adjacent to a device channel or (2.2) within a rod-patterned channel in the gel. In **A**) vertical MPS, a semipermeable membrane separates the tumor and endothelial compartments, providing anchorage for ECL adhesion. This image was created using BioRender (https://biorender.com/). Abbreviations: ECM, extracellular matrix; CAFs, cancer-associated fibroblasts

While the basic configuration of this ECM-focused design is primarily applied to model invasion, subsequent sections detail modified versions that have been engineered to replicate other stages of metastasis. For clarity, these systems are referred to as horizontal MPS due to the horizontal arrangement of their channels relative to the central chamber. This distinguishes them from vertical MPS [53, 55]which consist of two stacked compartments separated by a semipermeable membrane (Fig. 2) [20, 21, 40]. Both vertical and horizontal MPS are primarily made from polydimethylsiloxane (PDMS), widely used in microfluidic devices for its ease of fabrication and biocompatibility, though other materials may offer advantages depending on experimental needs [56]. While vertical MPS are typically limited to two compartments, usually one tumor and one endothelial (e.g., a chamber and a channel or two channels), horizontal MPS offer greater design versatility, incorporating multiple chambers that are either interconnected [17, 18, 35] or separated by media channels [8, 57, 58]. Moreover, some single-channel designs have also proven useful for investigating specific metastatic steps [37, 59–63]. Table 1 presents a compilation of MPS designs, along with details of their applications and the biological outcomes they have provided.

**Fig. 3:**
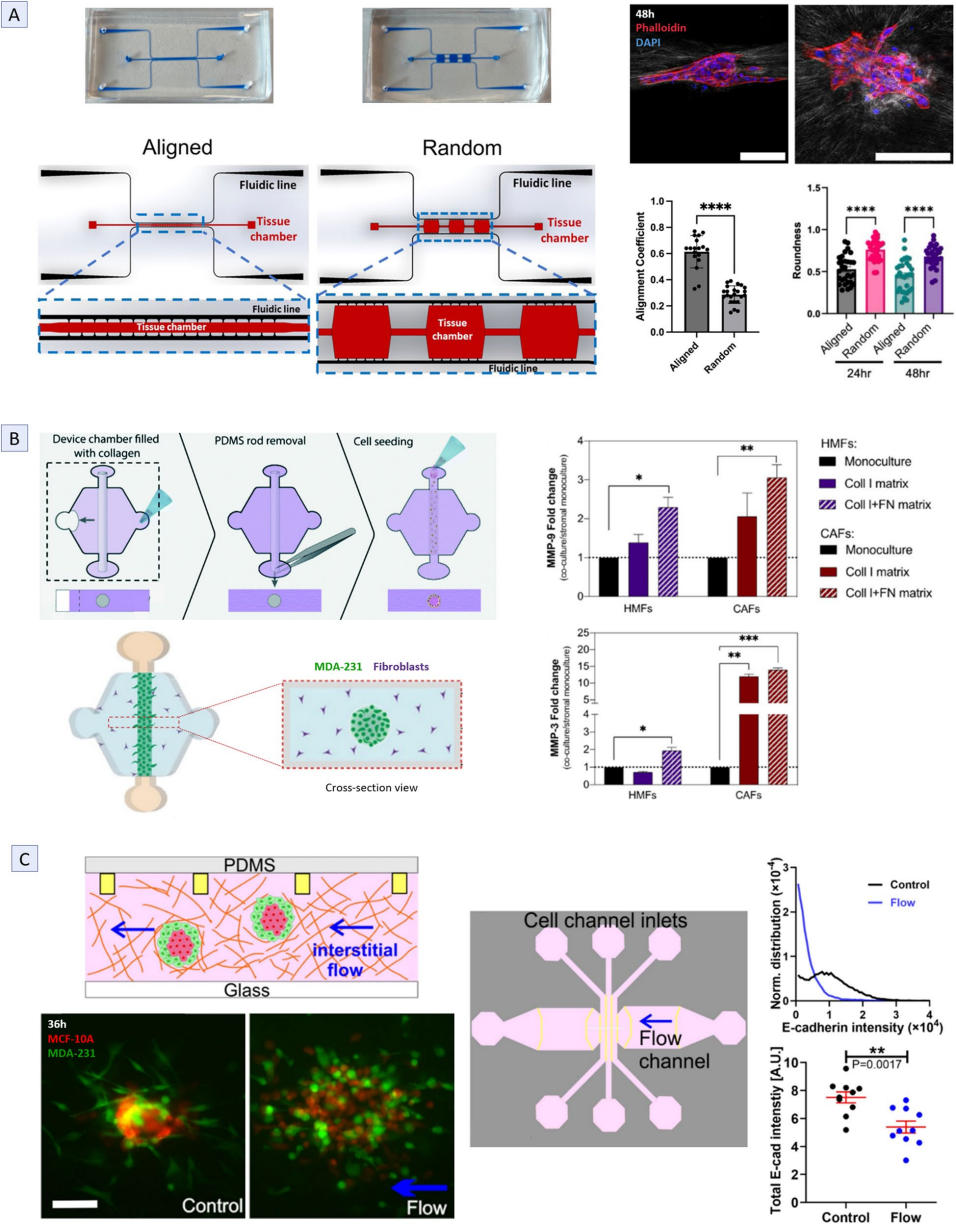
Representative MPS that mimic the invasion metastatic step. **A**) Horizontal MPS designs used to produce aligned and randomly oriented collagen fibers, accompanied by a graph quantifying fiber alignment coefficient in each device. Representative images from phalloidin (red) and DAPI (blue) staining at 48 h show organoid elongation in the direction of fiber alignment, supported by a graph of organoid roundness comparison between aligned and random devices; scale bar: 100 μm [64]. **B**) LumeNEXT horizontal MPS generated using the rod-patterned method to evaluate stromal influence on tumor invasion. The central chamber is filled with collagen I hydrogel embedded with fibroblasts and polymerized around a PDMS rod, which is later removed to create a hollow channel within the matrix, allowing for the filling with MDA-231 cells. Cross-sectional diagram illustrates cell distribution within the device. Graphs show MMP3 and MMP9 secretion from MDA-231 co-culture with human mammary fibroblasts (HMFs - purple) and cancer-associated fibroblasts (CAFs - red) in collagen (solid bar) or fibronectin (FN - striped bar) matrices. The fibroblast monoculture is represented by the solid black bar [72, 99]. **C**) Horizontal MPS containing collagen-embedded spheroids introduced through three inlet channels into the central chamber, where IFF can be generated via a perpendicular channel connected to a syringe pump. Representative images show spheroid disaggregation after 36 h with IFF (right) vs. control (left), caused by Ecad downregulation in MCF-10 A (red) and MDA-231 increased invasion (green) under fluidic stress; scale bar: 100 μm. Graphs depict Ecad distribution and intensity [9]. Statistical significance is indicated as follows: * for *P* ≤ 0.05, ** for *P* ≤ 0.01 and *** for *P* ≤ 0.001. (**A**) Copyright 2024, American Chemical Society, (**B**) Copyright 2020, MDPI and Royal Society of Chemistry, (**C**) Copyright 2020, Springer. Reproduced with permission

In most horizontal MPS, tumor cells are either seeded in one of the channels and invade the matrix toward a chemoattractant in the opposing channel (Fig. 2B) [49, 50]or embedded within the matrix in the central chamber as dispersed cells [18, 48] or self-assembled aggregates, like spheroids or organoids [34, 35, 64]. In contrast to vertical MPS, which feature physically separated compartments, horizontal MPS enable unhindered cell movement by reducing physical barriers. This is achieved through various design strategies, such as pillar-confined matrix compartments [29, 50, 65, 66]; pillarless designs that employ a surface treatment to modify the hydrophilicity of the materials, preventing matrix release until gelation [67–69]; or alternatively, designs compartmentalized by casting the gel around a needle or rod, which is removed after gelation to form hollow channels within the matrix [70–74]. An overview of the last method is illustrated in Fig. 3B.

By integrating ECM-mimicking matrices and chemotactic gradients into horizontal MPS platforms, researchers have successfully recapitulated invasion phenomena that were previously observable only in animal models [43, 76]. Among these, collective motility dynamics —where tumor cells move as cohesive groups, forming strands or sheets led by specialized leader cells³⁴,⁷⁶—has emerged as a particularly relevant mechanism. This coordinated mode of invasion is believed to enhance metastatic efficiency compared to single-cell invasion [12]. An investigation by Xia et al. in a horizontal MPS reproduced the typical cell organization and polarization of collective invasion in several cancer types — which was absent in traditional wound healing assays — and identified AURKA protein’s role in matrix metalloproteinase (MMP)-dependent invasion [50]. AURKA was expressed only in leader cells, and its overexpression was able to induce collective invasion in the non-invasive MCF-10 A cell line. Using an experimental approach where Matrigel in the central chamber separates cells in one channel from a serum chemoattractant in the other, the study provided valuable insights into the capabilities of leader cells, revealing AURKA as a potential therapeutic target to slow metastasis progression.

In collective invasion, cell movement is coordinated through stable or transient cell-cell and cell-ECM interactions, with E-cadherin (Ecad) playing a crucial role in maintaining connections between leader and follower cells [42, 43]. To examine this coordination, a single-channel MPS was designed and mounted on a fibronectin-coated cover glass, where channel walls were functionalized with either Ecad, to mimic lateral cell interactions, or pluronic, to inhibit adhesion [60]. In this setup leader cells were found to move faster in narrower, non-adhesive channels but often detached from the cluster. However, in Ecad-coated channels, cells remained cohesive, with leader cells using their cytoskeletal network to guide follower cells via Ecad-mediated junctions, supporting collective motility. The research concluded that while cell-ECM interactions control invasion speed, Ecad-mediated shear forces are essential for maintaining cluster cohesion, especially under strong traction forces. Although this study employed a non-cancerous epithelial cell line in a simplified 2D system, it helped to deliver important mechanistic insights about the importance of intracellular cohesion during collective invasion.

Previous studies have achieved collective invasion by densely seeding cells to form cellular aggregates. However, 3D self-assembled structures, such as spheroids or organoids, more accurately replicate the spatial organization found within primary tumors, exhibiting essential cell–cell interactions and biochemical gradients characteristic of dense tumor masses [77, 78]. As a result, these models offer substantially greater physiological relevance for investigating collective tumor behaviors, including leader–follower dynamics and cooperative matrix remodeling [63, 76, 79–81].

For example, a recent study investigated the invasion of MDA-MB-231 (MDA-231) spheroids embedded in collagen I within the central chamber of a horizontal MPS, exposed to a gradient of epidermal growth factor (EGF) [82]. Tumor cells were observed to emerge both as cohesive groups and as individual cells detaching from the invading front, reflecting the functional heterogeneity that can emerge within multicellular tumor aggregates. Compared to a previous study by the same group, in which cells were uniformly dispersed within the matrix [83]spheroid-derived cells exhibited significantly higher invasion velocities. This difference suggests that the spatial organization of tumor cells within aggregates may enhance invasive potential, possibly by promoting the emergence of leader-like phenotypes or facilitating synergistic interactions.

On the other hand, organoids offer increased biological complexity by comprising multiple cellular lineages and more accurately mimicking key structural and functional features of native organs [35, 64, 71]. Hwang et al. leveraged organoid heterogeneity to investigate the behavior of leader cells—defined by keratin 14 expression in breast cancer—under exposure to an SDF-1 chemokine gradient [35]. These cells were shown to upregulate receptors for SDF-1 in hypoxic conditions. By recreating a hypoxia-driven pro-invasive microenvironment using mouse mammary tumoroids embedded in a collagen I matrix within a multi-chambered horizontal MPS, the study demonstrated that leader cells relocated from random positions within the cluster to its invasive front, thus confirming their intrinsic phenotypic predisposition to lead collective invasion.

In addition to enabling the detailed study of chemotaxis-driven invasion, one of the key advantages of MPS is their ability to precisely control ECM characteristics such as stiffness, density, porosity, and fiber alignment—either through microfluidic design or by tuning the matrix source, concentration, or polymerization conditions. This versatility makes them a powerful platform for investigating how specific ECM alterations modulate tumor behavior. These alterations, considered an early and prominent hallmark of malignancy [5, 14]result from two synergistic mechanisms — active matrix remodeling via deposition and crosslinking by tumor and stromal cells, and passive fiber compression driven by tumor expansion [84, 85]. Together, these changes elevate mechanical stress, disrupt cellular mechanotransduction and promote tumor invasion [86], immune evasion and therapeutic resistance [5].

In the context of invasion, increased ECM stiffness and fiber disorganization have been shown to enhance tumor cell motility by affecting invasion speed, persistence, and the ability to invade collectivelly [87, 88]. Additionally, reduced matrix pore size may lead to cell confinement and deformation, forcing cells to adopt less efficient motility strategies [41, 65, 89, 90]. Consistently, most studies using collagen I matrices in MPS report that increased ECM stiffness hinders tumor cell invasion [48, 49, 65, 91], whereas matrices enriched with components for which cells have higher affinity can reverse this effect [65, 72, 92, 93]. In this regard, a study showed how non-small cell lung cancer (NSCLC) cells embedded in matrices with varying Matrigel-to-collagen I ratios exhibited faster motility in mixed matrices compared to collagen alone, even under conditions of increased stiffness [65]. This effect was attributed to enhanced MMP secretion induced by stiffness and greater cell affinity for Matrigel’s laminin, which facilitated the formation of focal adhesions [94]as well as to changes in matrix porosity and fiber organization. However, above a certain Matrigel concentration, excessive focal adhesions impaired cell movement, mitigating the previous effects. Using a horizontal MPS with H1299 cells seeded in the central chamber, the study underscored the delicate balance of integrin-mediated cell–ECM interactions, whereby higher matrix density promoted cell attachment but restricted invasion.

On the other hand, MPS are essential tools for investigating how fibrillar disorganization influences tumor cell invasion, as they offer precise control over collagen fiber alignment. Alignment can be modulated through various strategies, including chamber geometry modifications [93]coupling to strain-inducing devices [95]or by fine-tuning collagen polymerization parameters [96]. These models have consistently shown that tumor cells preferentially displace along aligned collagen fibers, where alignment enhances invasion efficiency by promoting directional persistence [95–97].

One noteworthy study induced a distinct fiber alignment pattern around a HeLa spheroid by inserting it, embedded in collagen, using syringe-pump: fibers became arranged tangentially around the sides of the spheroid and radially downstream [98]. A more recent study using mouse mammary tumoroids achieved comparable fiber alignment by altering chamber geometry—producing aligned fibers with a 20:1 length-to-width ratio and random orientation with a 1:1 ratio (Fig. 3A) [93].

While these platforms are highly valuable for dissecting specific mechanisms of ECM-guided invasion, they often lack the broader context of the TME, potentially overlooking the influence of other cellular and mechanical components—aspects addressed in the next section.

### Modelling the influence of stromal cells on invasion

Understanding how ECM changes influence invasion requires examining the role of stromal cells—particularly cancer-associated fibroblasts (CAFs), which represent the most prevalent and functionally impactful stromal population [7, 44]. While CAFs predominantly arise from tissue-resident fibroblasts activated by tumor-derived signals—driving a shift from tumor-suppressive to tumor-promoting phenotypes [5, 100]—they may also originate from mesenchymal progenitors, especially mesenchymal stem cells (MSCs), both local and bone marrow-derived, contributing to cellular heterogeneity of the TME [101, 102]. Their pro-invasive functions stem from their capacity to remodel the ECM by promoting both matrix synthesis and degradation, ultimately altering its composition, stiffness, and architecture to facilitate tumor cell migration and invasion [6, 14, 42].

Significant insight into these processes has been gained using MPS that allow co-culture of CAFs and tumor cells within the same scaffold [17, 72, 103]. These models also highlight the importance of reciprocal communication between both cell types during invasion, as evidenced by indirect co-culture approaches [101, 104] and conditioned medium experiments in MPS [30]which help clarify their signaling contributions. Beyond invasion, CAFs have also been shown to promote tumor cell proliferation [101, 102, 105]in contrast to healthy normal fibroblasts (NFs), which typically inhibit both tumor invasion and proliferation [72, 102, 103]. These systems also make it possible to study how biomechanical cues—such as IFF [8]shear stress [61]and contractile strain [30]— influence CAF-tumor interactions. Due to their genetic stability relative to tumor cells, CAFs are emerging as a promising therapeutic target for metastatic treatment [100].

One key mechanism by which CAFs have been shown to promote tumor invasion is through the upregulation of MMP (MMP3 and MMP9), a behavior that was not observed in NFs unless fibronectin was present in the matrix (Fig. 3B) [72]. Such differential MMP regulation explains the minimal differences in MDA-231 invasion when co-cultured with either fibroblast type in fibronectin-rich matrices, emphasizing how tumor-altered ECM composition can drive metastasis. These findings were facilitated by an innovative LumeNEXT pillarless horizontal MPS design [70, 71], which employs the rod-patterning method to recreate a lumen structure within the gel in the central chamber. This system enables co-culture of MDA-231 tumor cells filling the lumen and collagen-embedded fibroblasts surrounding it, providing a physiologically relevant model for studying tumor-stroma interactions (Fig. 3B). Investigating the contribution of CAFs heterogeneity to tumor progression, Nikkhah’s group demonstrated that CAFs derived from breast cancer subtypes with varying aggressiveness exert distinct effects on tumor cell invasion and proliferation, while primary NFs consistently exhibited tumor-suppressive effects, even reducing invasion compared to monoculture controls [103]. Interestingly, when analyzing CAFs motility, which were mostly stationary in the absence of tumor cells, enhanced invasion was observed when co-cultured with highly aggressive tumor cells [8, 103]. This bidirectional interaction mirrors fibroblast recruitment during physiological wound healing, a process frequently exploited by the tumor to promote progression [5, 6]. These observations were made using a concentric-layered horizontal MPS in which tumor cells were cultured in a central chamber surrounded by a stromal chamber containing collagen-embedded fibroblasts.

Later, another MPS was developed with separate chambers for tumor cells and fibroblasts, connected by zigzag channels to deepen the investigation of tumor–CAF crosstalk [101]. This design allowed unidirectional communication along the X-axis and the establishment of a gradient of signaling molecules along the Y-axis across the Matrigel matrix. Human MSCs and MRC-5 lung fibroblasts were used as stromal cell sources to differentiate to CAFs. The study revealed that aggressive tumor cell lines, such as MDA-231, induced CAF activation more effectively than less metastatic lines like MCF-7, correlating with increased transforming growth factor β (TGF-β) release. Inhibition of TGF-β, achieved via an annexation channel that blocked flow to the stromal chamber, completely neutralized CAF activation, confirming its pivotal role in this interaction. Additionally, fibroblast-released TGF-β acted as a key pro-tumoral signal, enhancing tumor cell invasion, proliferation, and spontaneous spheroid formation within the tumor compartment.

Alongside CAFs, tumor-associated macrophages (TAMs) are a major stromal component that actively remodels the ECM and engages in reciprocal crosstalk with tumor cells [5]. Derived from circulating monocytes, they differentiate into macrophages that adopt distinct functional states in response to local cues [106, 107]. The direction of this polarization critically shapes their impact on invasion: M1-like TAMs exert anti-tumor effects, whereas M2-like TAMs promote metastasis by secreting pro-angiogenic factors, immunosuppressive cytokines, and ECM-remodeling enzymes [44, 66].

During tumor progression, TAM population shifts the phenotype balance toward M2, reinforcing the pro‑tumorigenic microenvironment. This shift has been confirmed in several studies using horizontal MPS, where co-culture of tumor cells with macrophages drives stable M2 differentiation via colony-stimulating factor 1 (CSF-1) signaling [66, 108, 109]. In one of these studies, M2-TAMs, in response, enhanced MDA-231 cell invasion and reduced paclitaxel-induced cytotoxicity, highlighting their dual role in promoting invasion and chemoresistance [66].

Based on this, Bi et al. developed a more complex tumor–TAM interaction model, in which tumor cells and macrophages were loaded into two matrix chambers flanking a central microvascular network chamber [106]. They showed that M1-like TAMs suppressed tumor proliferation, motility, and angiogenesis, while M2-like TAMs significantly promoted invasion toward the vasculature without compromising endothelial integrity. Further expanding these findings, Gadde et al. investigated the effect of M2 TAMs on ECM architecture and vascular barrier function [107]. Using a horizontal MPS with a rod-patterned endothelial lumen surrounded by tumor cells and TAMs embedded in a collagen matrix, they found that M2 TAMs increased ECM porosity and vascular permeability, facilitating both invasion and intravasation into the vascular network.

### Modelling the influence of interstitial fluid flow on invasion

Local ECM stiffening and compression caused by tumor expansion contribute to elevated interstitial fluid pressure, creating a pressure imbalance between cancerous and healthy tissue that drives IFF at the periphery of the tumor [14]. This effect is usually exacerbated by fluid accumulation from vascular leakage and impaired lymphatic drainage, both of which are common abnormalities in the primary TME [15, 16]. Recently, interstitial fluid pressure and IFF have emerged as critical factors in cancer progression and therapeutic resistance. These forces regulate the distribution of nutrients, signaling molecules, and drugs in healthy tissues while also modulating tumor cell behavior and activating immune and stromal cells within the TME [15, 110–112].

While early insights into the role of IFF in tumor invasion emerged from modified Boyden chambers—revealing two complementary mechanisms by which IFF promotes invasion, namely convective cytokine transport and the direct influence of fluid shear stress tumor mechanosensing [113]—recent advancements in perfusable MPS have significantly propelled this field by enabling finely tunable IFF within the ECM-like hydrogels with greater spatial and temporal precision [110]. Building on the horizontal MPS design, these systems incorporate one or more matrix chambers, perfused from one side to the other via lateral channels [35, 109, 114, 115] or through a channel that crosses the central chamber perpendicularly (Fig. 3C) [9]. Basic setups generate passive IFF through hydrostatic pressure gradients from media height differences and/or asymmetric channel designs, sustaining these conditions for several hours without the need for external actuation [8, 71, 116]. For applications requiring more precise control or continuous flow, more advanced configurations involve the use of microfluidic pumps connected to the channels of the device, which include syringe pumps [9, 93, 101, 102, 117] peristaltic pumps [36, 40, 59, 118] and pressure-based pumps [37, 38, 114] depending on culture and experimental requirements.

The effect of IFF on tumor cell motility and migration direction has been demonstrated in several studies using MPS [9, 34, 35, 117, 119, 120]. In a landmark study, Polacheck et al. used a horizontal MPS to apply IFF across a central tumor chamber via a hydrostatic pressure gradient [120]. They showed that MDA‑231 cells migrate upstream IFF through a mechanism driven by integrin-mediated activation of FAK, uncovering a chemokine-independent pathway by which fluid forces guide tumor cell invasion in 3D environments.

Building on these foundational insights, more recent studies have shifted focus towards exploring the effect of IFF on multicellular aggregates such as tumor spheroids [9, 93, 121]. One such study by Huang et al. investigated the effect of IFF on heterotypic spheroids embedded in a collagen I matrix, used to model breast cancer architecture. In this setup, perfusion was incorporated through a channel perpendicular to the central chamber of a horizontal MPS (Fig. 3C) [9]. As a result, IFF was found to induce spheroid dissociation by disrupting Ecad junctions in non-tumorigenic MCF-10 A cells and to shift MDA-231 motility from mesenchymal to amoeboid, a mode previously shown to be faster in this cell type [34]. In contrast, under static conditions, non-tumorigenic cells remained within the spheroid core, while only peripheral MDA-231 cells invaded the matrix, thus recapitulating the invasive behavior of ductal carcinoma. As an alternative design, a vertical MPS with honeycomb porous supports coated with either collagen I or BM-like matrix was used [93]. When ovarian cancer spheroids were exposed to IFF perfused beneath the channel, IFF selectively promoted invasion in a matrix-dependent manner, enhancing single-cell invasion in collagen I, while having minimal effect in BM matrix [93].

Beyond its direct effect on tumor cells, IFF critically contributes to cell–cell communication within the TME by enhancing the transport of secreted factors [15, 122]. This role was demonstrated in a study using an innovative horizontal MPS design featuring two tumor channels flanking a central fibroblast channel, separated by two matrix compartments [8]. By generating a pressure gradient across the outer channels, the system produced IFF toward the central CAF compartment, enabling the transport of cytokines from NCI-H28 mesothelioma cells and thereby promoting CAF activation and motility.

Expanding on this concept, several studies using horizontal MPS have revealed a synergistic interaction between IFF and tumor cells in driving TAM polarization toward the M2 phenotype [108, 109, 115]. Notably, one study observed that TAMs migrate upstream IFF, similarly to tumor cells, as indicated by actin accumulation on their flow-facing side, suggesting that IFF may also contribute to TAMs recruitment toward the primary tumor [109].

Although invasion-focused MPS allow the modeling of specific processes not feasible in conventional cultures—such as chemotaxis, fiber alignment, or IFF-driven migration—their use as standalone models is limited. Most platforms currently addressing invasion also incorporate vascular components, often coupling this process with intravasation, reflecting a broader shift toward more comprehensive models. This emphasis has contributed to the underrepresentation of certain components of the primary TME, such as less-explored mechanical cues (e.g., cyclic deformations or compression from tumor growth) and specific stromal populations, including undifferentiated MSCs. Altogether, these trends underscore the ongoing transition toward vascularized systems, which are discussed in the following section.

## Microphysiological systems for studying vascular-related metastatic steps

Tumor growth leads to nutrient deprivation and hypoxia in the core, triggering angiogenic responses in nearby blood vessels to restore nutrient supply [14, 44]. However, conflicting signals within TME often result in abnormal vascularization, further worsening the nutrient-deprived conditions [16]. This vicious cycle accelerates tumor cell progression through the tissue, facilitating their access to the vasculature and promoting intravasation [24, 123]. Once tumor cells cross the vascular or lymphatic endothelium, they travel through the systemic circulation as either individual circulating tumor cells (CTCs) or as clusters [124]. Upon arrest at a distant site — mediated by passive or active factors — they can extravasate into the target organ microenvironment, where they may establish and proliferate, forming a secondary tumor niche [19, 31].

Tumor cell invasion into the stroma, tumor-induced angiogenesis and intravasation are interconnected and occur simultaneously at the onset of metastasis [7, 125]. Due to their complexity, studying these processes requires a comprehensive analysis of the TME factors involved and their downstream effects. In this context, MPS models are highly valuable for replicating the microscale features of the human vascular network and enabling high-resolution monitoring of tumor-vascular dynamics [38, 58, 126] a task that remains challenging in animal models and unreliable in traditional in vitro systems. Consequently, several studies have demonstrated that MPS more effectively recapitulate intravasation and extravasation events, as well as the heterogeneous phenotypes of CTCs compared to the Boyden chamber [29, 30].

Despite occurring in distinct environmental scenarios, both intra- and extravasation models share a common basis: reproducing tumor cell interactions with the vasculature to replicate their traversal across the endothelium. In intravasation models, tumor cells invade the matrix and cross the endothelium to enter the circulation [17, 18, 71], whereas in extravasation models tumor cells are perfused through the endothelium, where they adhere to and traverse the vascular barrier to colonize the secondary stroma [38, 75, 114, 116]. The intermediate step, circulation, is typically modeled using single-channel MPS or micro-patterned constriction arrays that lack endothelialization [37, 127, 128].

There are two primary methods for vascularizing MPS. The first, termed endothelial cell lining (ECL), involves adhering endothelial cells to the walls of a channel to simulate a tubular vessel (Fig. 2A). This can be achieved either within a channel separated by a semipermeable membrane in vertical MPS [30, 75, 102] or within a tubular cavity generated in the matrix of a horizontal MPS [73, 92] (Fig. 2B). The second method entails the formation of a microvascular network (MVN) through vascular self-assembly and is typically implemented within the matrix chamber of horizontal MPS [18, 38, 57, 116] (Fig. 2B). In this approach, endothelial cells are embedded in a fibrin-based hydrogel, often alongside fibroblasts to promote microvascular development [17, 105]. Human umbilical vein endothelial cells (HUVECs) are commonly used for constructing blood vessels, while human lymphatic endothelial cells (HLECs) are used to recreate the more permeable nature of lymphatic vessels.

After outlining the most common endothelialization strategies, the comparison between horizontal and vertical MPS can be further developed by incorporating the distinction between ECL and MVN. When focusing on ECL over a membrane in vertical systems and MVN within horizontal platforms, vertical MPS offer key advantages, such as the ability to recreate a more controlled and reproducible vascular geometry, assess endothelial polarity-dependent processes, and study transendothelial molecular transport [110]. On the other hand, their configuration may hinder high-resolution imaging, and the presence of a semipermeable membrane introduces a physical barrier, making these systems less suitable for evaluating tumor cell crossing of the endothelial barrier.

In contrast, MVNs implemented in horizontal MPS benefit from a planar design that enhances imaging capabilities, more closely mimic native vascular development, incorporate a stromal environment that supports 3D tumor invasion, and enable direct interaction between the endothelium and the tumor. Despite these advantages, the irregular anatomy of MVNs limits their ability to sustain biologically relevant and uniform shear stress levels, which remains a primary strength of ECL approaches in vertical MPS [129]. Vascular shear stress is essential for maintaining endothelial normal function under controlled conditions and represents a key mechanical constraint for tumor cell progression during vasculature-related metastatic steps [130].

Notably, ECL of a rod-patterned tubular cavity within the matrix of horizontal MPS combines the geometric control and shear stress benefits typically associated with vertical systems, with the 3D microenvironment and vascular remodeling potential of MVNs. These features make horizontal MPS the most widely adopted platforms for investigating vasculature-mediated steps of metastasis. Focusing on horizontal MPS, the main advantages of MVNs over the ECL method include a more physiological representation of vascular structure and remodeling. Although they introduce greater variability, this architecture additionally allows for the study of tumor cell arrest at the endothelium driven by vascular bifurcations or low-flow regions [131].

### Modelling tumor cell intravasation

Intravasation is an unpredictable event whose likelihood is strongly influenced by the proximity of the tumor to the vasculature and the structural integrity of the vessels [16, 31]. Tumor-vessel proximity is often enhanced by crosstalk between tumor and endothelial cells in response to microenvironmental signals, which promote mutual approach through growth and motility mechanisms. Studies using MPS have demonstrated enhanced angiogenesis [17, 71] and increased tumor invasiveness and proliferation [18, 59, 105] in tumor–endothelial co-culture models.

Beyond spatial proximity, the status of the endothelial barrier also plays a critical role. In the TME, elevated pro-inflammatory factors can compromise vascular permeability, thereby facilitating tumor cell intravasation [132, 133]. Notably, this increased permeability can be strategically exploited in nanomedicine to selectively deliver therapeutic agents to the TME, maximizing treatment efficacy while minimizing systemic toxicity, a phenomenon known as the enhanced permeability and retention (EPR) [134, 135].

**Fig. 4:**
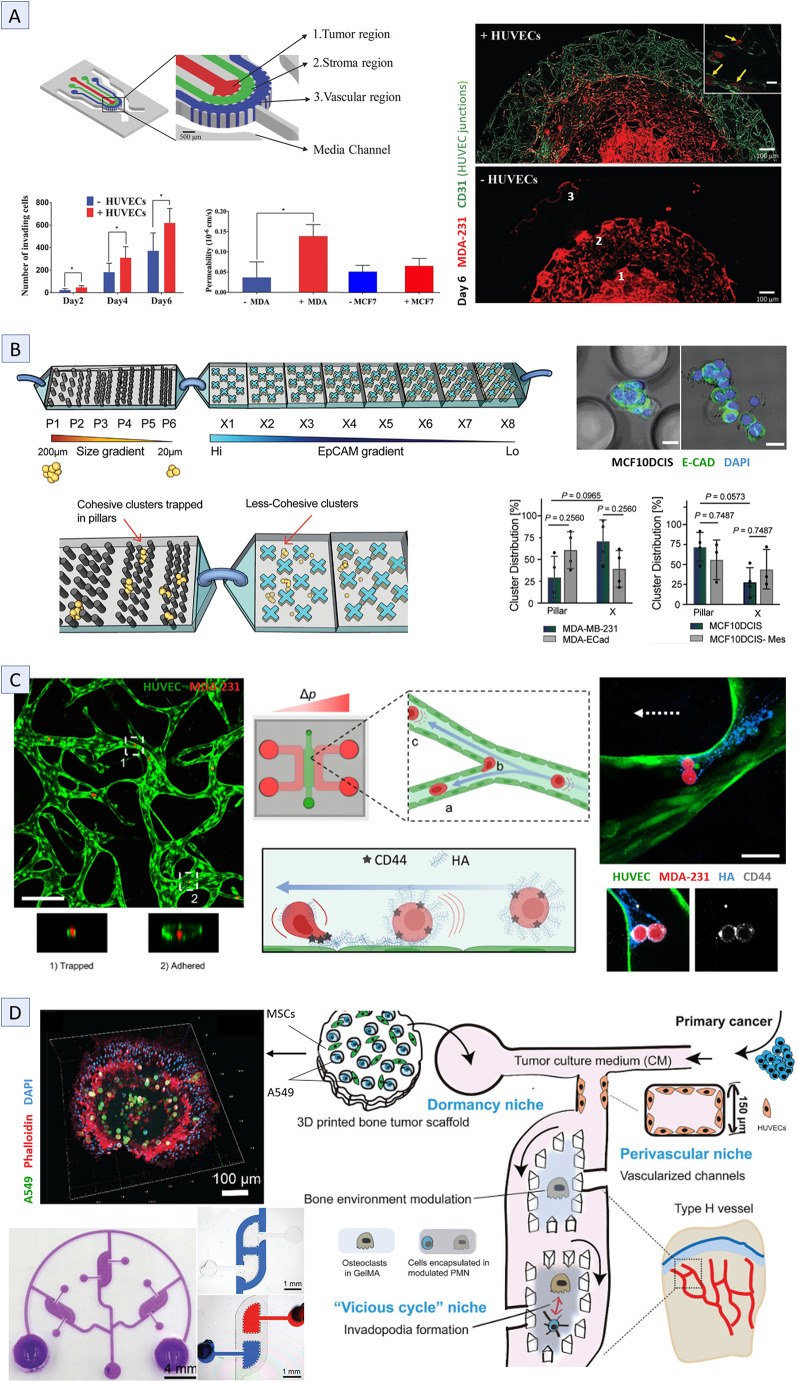
Representative MPS that mimic vascular-related metastatic steps and colonization. **A**) Concentric layered horizontal MPS used to study tumor cells intravasation. Representative images of MDA-231 cell (red) invasion into the device compartments: (1) tumor, (2) stromal, (3) vascular, with (top) and without (bottom) HUVECs on day 6. HUVECs are stained with CD-31 (green) for cell junctions. Yellow arrows indicate intravasated cells. Scale bar: 100 μm. Graphs show the number of invading MDA-231 cells in the stromal chamber depending on the presence of HUVECs, and endothelial permeability (FITC-dextran 70 kDa) in co-culture with MDA-231 or MCF-7 cells [18]. **B**) PilarX array of microconstrictions MPS for profiling circulating tumor cells based on physical properties and EpCAM expression. A zoomed-in view of compartments P4–P6 of the Pillar-device and X1–X2 of the X-device. Immunostaining of MCF10DCIS clusters trapped in P5 (left) and X1 (right); scale bar: 20 μm. Graphs display cluster distribution in each device for MDA-231 and MCF-10DCIS cells compared to their genetically modified counterparts (MDA-Ecad and MCF-10DCIS-Mes) [139]. **C**) Horizontal MPS with an MVN in the central chamber, used to investigate the role of endothelial glycocalyx in tumor cell adhesion prior to extravasation. In this setup, tumor cells are perfused under a pressure gradient (Δp), generating a luminal flow that transports them until they become arrested through adhesion or entrapment in (a) narrow channels or (b) capillary bifurcations. Representative image (left) of MDA-231 cells (red) arrested within the MVN (green) by various mechanisms; scale bar: 200 μm. Diagram illustrates glycocalyx-mediated tumor cell adhesion via hyaluronic acid (HA) deposition and CD44 anchoring (arrow indicates flow direction), accompanied by a confocal image showing tumor cells attached to hyaluronic acid streaks (blue) through their CD44 receptors (gray); scale bar: 60 μm [38]. **D**) Horizontal MPS featuring distinct BME niches connected by endothelialized H-shaped channels to study dormancy reactivation in lung cancer. Images of dyed channels demonstrate independent fluid flow in channels and chambers; scale bar: 1 mm. Confocal image of the dormancy niche shows lung cancer A549 cells (green) and MSCs co-culture; scale bar: 100 μm [39]. Statistical significance is indicated as follows: *P ≤ 0.05, **P ≤ 0.01, ***P ≤ 0.001. (A) Copyright 2018, Wiley, (B) Copyright 2022, Wiley, (C) Copyright 2021, Springer, (D) Copyright 2023, Wiley. Reproduced with permission

This interplay between the tumor and its surrounding vasculature leading to tumor cell intravasation has been well illustrated in several MPS designs. Using an MVN model on a horizontal MPS comprising three gel chambers (tumor cells and endothelial cells on the sides, CAFs in the middle), Zhang et al. observed that MDA-231 cells increased their invasive distance toward the endothelium, while inducing angiogenesis by promoting vessel sprouting from the MVN to facilitate their intravasation [17]. Another study, by Nikkhah’s group, using the previously developed concentric layered horizontal MPS [103], found that only the aggressive MDA-231 cells invaded the stromal chamber, disrupted vascular permeability and intravasated the MVN, while less invasive MCF-7 cells showed no such behavior (Fig. 4A) [18]. An alternative approach used a vertical MPS with an array of cylindrical tumor compartments, containing Matrigel-encapsulated MDA-231 cells and fibroblasts, connected by a syringe pump-perfused top endothelial channel [102]. This configuration allowed the observation of tumor cells crossing the endothelium into the upper channel, while endothelial cells migrated into the tumor compartment and formed self-assembled spheroids with the tumor cells.

Despite extensive research on tumor-vessel interactions during intravasation, the precise mechanisms behind this process remain poorly understood. Transendothelial migration (TEM) is widely considered a primary pathway for tumor cells to traverse endothelial adhesions or transient focal leaks [31, 125]. However, the underlying biophysical and physiological processes — such as alterations in cell polarity, cytoskeletal and nuclear architecture, and membrane receptor expression — have yet to be fully elucidated. In an investigation using ECL vessels on rod-patterned gel channels of a horizontal MPS, Wong and Searson identified the role of mitosis as a mechanical driver of tumor cell intravasation [126]. Microscopic analysis revealed that mitotic cell rounding on the endothelial wall generated a mechanical force that transiently disrupted focal adhesions, exposing tumor cells to shear flow, which then drew the daughter cells into circulation. During this process, invasive cells extended protrusions into the ECM-endothelium interface, and upon mitosis, their increased circularity deformed the endothelium more easily than the stiff matrix. When this deformation was sufficient to open endothelial junctions, the daughter cells became exposed to the vessel lumen, where shear stress facilitated their entry into circulation. In contrast, extending protrusions between endothelial junctions without mitosis caused cell rupture. The involvement of mitosis in intravasation was further evidenced by a dramatic reduction of this event following treatment with the anti-proliferative drug DXR.

When a primary tumor lies in close proximity to a blood vessel, its cells can directly interact with the endothelium, bypassing the EMT shift and ECM traversal that typically precede intravasation in the traditional linear cascade model of metastasis [43, 125]. In a follow-up work, Searson’s group investigated this phenomenon using a horizontal MPS, where matrix-integrated breast cancer tumoroids, simulating primary tumors, interacted with endothelial cells lining a hollow channel within the gel [71]. The authors found out that tumoroids located close to the vascular channel were able to infiltrate through leaky endothelial junctions, forming mosaic vessels. These structures were then fragmented by flow-induced shear stress, releasing individual CTCs or clusters into the channel. While mosaic vessel formation was the predominant interaction, two other intravasation mechanisms were identified: organoid wrapping around the vessel, constricting its lumen, and organoid pulling on the microvessel, displacing it. In addition to providing a detailed view of the intercellular dynamics between the tumor and its surrounding vasculature — which may help explain the abundance of tumor aggregates in the circulation — the study identified two mechanisms that may contribute to the vascular abnormalities observed in vivo, including hypoxic regions and vascular co-option [16].

While most metastasis studies focus on intravasation into the bloodstream, it is still widely believed that epithelial cancers primarily metastasize through lymphatic vessels, which have weaker intercellular junctions and experience lower shear stress compared to blood vessels [24, 125]. Despite the numerous studies on lymphatics performed in MPS, most of them examine lymphatic dysfunction in isolation [136–138] often overlooking the impact of tumorigenesis on lymphatic integrity [70]. In another study by Lugo-Cintrón et al., the LumeNEXT MPS was used to evaluate how matrix stiffening affects lymphatic function and tumor cell behavior [73]. By comparing low and high-density collagen matrices mimicking healthy and cancerous breast tissue, the researchers found that HLECs cultured in the high-density matrix showed increased lymphatic leakage due to elevated interleukin (IL)-6 secretion. Changes in vessel morphology, actin stress fibers, and cell proliferation were also observed. This effect was potentiated by co-culturing with MDA-231 tumor cells, which further enhanced IL-6 secretion in the high-density matrices.

**Table 1:** MPS experimental approaches for mimicking metastatic steps

Cancer type and culture system	Other cell types	MPS and experimental design	Biological outcome	Ref
	A) Native stromal cells B) Endothelial cells C) Immune cells D) Host organ cells	A) MPS setup B) Matrix C) Flow D) Vascularization method		
**Metastasis step: Invasion (1)**				
*Influence of the Extracellular Matrix (ECM)*				
MCF10A healthy breast or MDA-MB-231 breast cancer cells seeded in the channel, on the side of the matrix		A) Horizontal MPS with a central chamber, flanked by two channels B) Matrigel	• Identification of the role of the AURKA protein in the leader cells of the collective cluster.	[50]
Mice mammary tumoroids embedded in matrix		A) Horizontal MPS with three central chambers, flanked by two channels B) Collagen I C) Hydrostatic pressure	• Leader cells orchestrate collective migration toward chemoattractant gradients from front positions.	[35]
Mice mammary tumoroids and MDCK epithelial cysts embedded in matrix		A) Horizontal MPS with a central chamber of variable geometry, flanked by two channels B) Collagen I C) Hydrostatic pressure	• Aligned matrices enhance elongation of tumoroids and epithelial cysts, guiding migration along fibers. • Fiber orientation supports morphogen gradient effects on epithelial cysts.	[64]
H1299 lung cancer cells embedded in matrix		A) Horizontal MPS with a central chamber, flanked by two channels B) Collagen I and Matrigel	• Denser ECM impedes tumor cell invasion, while optimal Matrigel promotes motility via MMP secretion and focal adhesion strengthening.	[65]
*Influence of Stromal Cells: Cancer-Associated Fibroblasts (CAFs)*				
MDA-MB-231 breast cancer cells seeded within a hollow gel channel	A) CAFs and mammary NFs	A) Horizontal MPS with a central chamber traversed by a rod-patterned channel B) Collagen I and fibronectin-enriched collagen	• CAFs increase MMP3/9 secretion in response to tumor signaling, while NFs do so only in fibronectin-rich matrices.	[72]
SUM-159 breast cancer cells embedded in matrix in the central chamber	A) CAFs from different breast cancer subtypes and NFs	A) Horizontal MPS with concentric layers of chambers and an external channel B) Collagen I and Matrigel	• CAFs from more aggressive breast cancer subtypes exhibit a stronger effect on invasion and proliferation of tumor cells. • NFs suppressive effect reduces tumor inherent invasive capacity. • CAFs become more invasive when co-cultured with tumor cells.	[103]
MDA-MB-231 or MCF-7 breast cancer cells embedded in matrix in tumor chamber	A) MRC-5 lung fibroblasts and mesenchymal stem cells (MSCs)	A) Horizontal MPS with two chambers connected by zig-zag channels B) Matrigel C) Syringe pump	• Identification of the role of TGF-β as the key signaling molecule mediating CAF’s activation by tumor cells. • Aggressive tumor cell lines release more TGF-β, activating more CAFs.	[101]
*Influence of Interstitial Fluid Flow (IFF)*				
MDA-MB-231 and MCF-7 breast cancer heterotypic spheroids embedded in matrix		A) Horizontal MPS with a central chamber traversed by a perpendicular channel B) Collagen I C) Syringe pump	• IFF disrupts Ecad, driving spheroid dissociation in MCF-10 A cells. • IFF promotes amoeboid motility in MDA-MB-231 cells, enabling faster migration. • Under static conditions, MCF-10 A cells remain in the core, while only peripheral MDA-MB-231 cells invade the matrix.	[9]
SKOV-3 ovarian cancer spheroids seeded on matrix-coated supports facing the channel		A) Vertical MPS separated from the channel by custom honeycomb support B) Collagen I and mixed composition BM matrix C) Syringe pump D) -	• Collagen I matrices promote individual cell migration, while collective migration dominates in BM matrix. • In collagen matrices, IFF increases leader cell mobility and spheroid shrinkage.	[93]
NCI-H28 mesothelioma cells seeded in the external channels	A) LL-24 lung fibroblasts	A) Horizontal MPS with three cell channels separated by two matrix chambers B) Collagen I C) Hydrostatic pressure	• IFF enhances transport of signaling cytokines from tumor cells, boosting CAFs motility.	[8]
**Metastasis step: Intravasation (2)**				
MDA-MB-231 breast cancer cells embedded in matrix in one of the chambers of the device	A) Human mammary CAFs B) HUVECs	A) Horizontal MPS with three central chambers, flanked by two channels B) Fibrinogen-based C) – D) MVN	• Tumor cells induce angiogenesis by promoting sprouting in the MVN, while also increasing invasion and intravasation when co-cultured. • DXR combined with apatinib is most effective against tumor cells, also enhancing vascular damage in the MVN. • CAFs are essential for MVN formation and maturation.	[17]
MDA-MB-231 or MCF-7 breast cancer cells embedded in matrix in the central chamber	A) - B) HUVECs	A) Horizontal MPS with concentric layers of chambers and an external channel B) Fibrinogen-based C) - D) MVN	• MDA-MB-231 cells can invade the stromal chamber, disrupt vascular permeability, and intravasate the MVN, unlike the less invasive MCF-7 cells.	[18]
MDA-MB-231 breast cancer cells embedded in matrix around a hollow gel channel	A) - B) HUVECs	A) Horizontal MPS with a central chamber traversed by a rod-patterned channel B) Collagen I with fibronectin coating C) Hydrostatic pressure D) ECL	• Identification of mitotic rounding as a mechanical driver for the disruption of endothelial focal adhesions, facilitating intravasation. • DXR treatment revert this effect.	[126]
Human and mice mammary tumoroids embedded in matrix around a hollow gel channel	A) - B) HUVECs	A) Horizontal MPS with a central chamber traversed by a rod-patterned channel B) Collagen I with fibronectin coating C) Hydrostatic pressure D) ECL	• Tumoroids near the vascular channel integrate into the endothelium, forming mosaic vessels that can be released into circulation under flow. • Identification of two other interactions: vessel wrapping and pulling.	[71]
MDA-MB-231 breast cancer cells embedded in matrix around a hollow gel channel	A) - B) HLECs	A) Horizontal MPS with a central chamber traversed by a rod-patterned channel B) Fibrinogen-based C) - D) ECL	• Higher density matrix promotes lymphatic leakage by inducing IL-6 secretion compared to low density matrix. • Co-culture with tumor cells further exacerbates this effect.	[73]
**Metastasis step: Circulation (3)**				
Perfused MDA-MB-231 and SK-BR-3 breast cancer cells		A) Single-channel MPS of varying geometries and evenly spaced microconstrictions B) - C) Pressure-based pump	• Perfusion and constriction-induced CTC deformation cause higher DNA damage and repair in SK-BR-3 cells compared to MDA-MB-231, where these mechanisms are constitutively active. • SK-BR-3 cells deformation causes EMT upregulation and larger nuclear size.	[37]
Perfused MDA-MB-231 and MCF-7 breast cancer cells		A) Array MPS of microconstrictions of varying sizes B) - C) Syringe pump	• At low pressures, CTC deformation is cytoplasm-driven; at higher pressures, it relies on nuclear stiffness. • MDA-MB-231 translocate more than MCF-7 due to their smaller and softer nuclei. • Translocated MCF-7 undergo a reduction in nuclear size and stiffness.	[128]
MDA-MB-231 and MCF-10ADCIS breast cancer clusters, pure and genetically modified		A) Array MPS of microconstrictions and functionalized magnetic nanoparticles B) - C) Syringe pump	• Clusters with mixed epithelial and mesenchymal features translocate the most efficiently due to their stability and flexibility.	[139]
**Metastasis step: Extravasation (4)**				
Perfused MDA-MB-231 breast cancer cells		A) Horizontal MPS with a central chamber, flanked by two channels B) Fibrinogen-based C) Pressure-based pump D) MVN	• Luminal flow significantly increases CTC extravasation compared to static conditions. • Trans-endothelial flow primarily affects subsequent invasion into the matrix.	[114]
Perfused MDA-MB-231 breast cancer cells		A) Horizontal MPS with a central chamber, flanked by two channels B) Fibrinogen-based C) Pressure-based pump D) MVN	• Identification of CTCs arrest mechanisms within the MVN. • CTCs deposit HA onto exposed endothelial cells, mediating adhesion via the CD44 receptor. • CD44 expression correlates positively with extravasation efficiency and is overexpressed in malignant cell lines.	[38]
Perfused SK-BR-3 breast cancer cells	A) – B) HUVECs and HLECs	A) Horizontal MPS with a central chamber, flanked by two channels B) Matrigel C) Syringe pump D) ECL	• Circulating SK-BR-3 cells adhere and cluster in the lymphatic channel before extravasating into the matrix. • IL-6 stimulation induces cluster elongation. • VEGF released by HLECs promotes angiogenesis from the vascular channel.	[75]
Perfused A375, A375-MA2 melanoma cells, and MDA-MB-231 breast cancer cells	A) – B) HUVECs C) Neutrophils	A) Horizontal MPS with 8 connected central chambers flanked by two channels B) Fibrinogen-based C) Hydrostatic pressure D) MVN	• Inflammatory neutrophils aggregate with CTCs, increasing their arrest through physical trapping and endothelial adhesion. • Aggregated neutrophils elevate IL-8 secretion, disrupting endothelial unions.	[116]
MDA-MB-231 breast cancer cells seeded on the endothelium	A) – B) Human dermal microvascular ECs C) Monocytes	A) Horizontal MPS with three cell channels separated by two matrix chambers B) Collagen I C) Hydrostatic pressure D) ECL	• Circulating monocytes enhance CTCs extravasation via MMP-9 secretion. • Extravasated monocytes differentiate into macrophages, whose movement creates microtracks that facilitate tumor cells invasion.	[58]
**Metastasis step: Colonization (5)**				
HCT116 colorectal cancer seeded in photopatterned holes in matrix	A) – B) HUVECs C) - D) Liver (HepG2) and lung cancer (A549) cells	A) Multi-chamber MPS with 4 parallel chambers equidistant to a central one B) Hyaluronic acid-based C) Peristaltic pump D) -	• CTCs initially adhere and proliferate in the lung niche, followed by the liver, mirroring their native organotropic preference.	[36]
Perfused MDA-MB-231 breast cancer cells	A) - B) Liver sinusoidal ECs C) - D) Liver epithelial (THLE-2) cells and lung fibroblasts	A) Vertical MPS with two channels separated by a porous membrane B) Matrigel C) Hydrostatic pressure D) ECL	• Tumor EVs induce EndMT in ECs, disrupting the barrier. • Preexposure to tumor EVs increases CTC adhesion by upregulating fibronectin via TGF-β, in a cell-type specific manner. • TGF-β activates fibroblasts and drives hepatic differentiation, aiding liver pre-metastatic niche formation.	[20]
MDA-MB-231 breast cancer, T24 bladder cancer and OVCAR-3 ovarian cancer cells seeded on the endothelium	A) – B) HUVECs C) – D) hBM-MSCs	A) Horizontal MPS with a central chamber, flanked by two channels B) Collagen I C) – D) ECL	• Bone signaling enhances CTC extravasation compared to acellular matrices. • The device captures distinct bone tropism among different cancer cell lines.	[140]
A549 lung cancer cells embedded in matrix	A) - B) HUVECs C) - D) Mouse osteoclasts (RAW 264.7) and MSCs	A) Horizontal MPS with two chambers connected by an H-shaped channel B) HAP and GelMA C) Syringe pump D) ECL	• Osteoclast-induced factors reactivate tumor dormancy, as evidenced by enhanced invadopodia formation, while MSCs maintain a dormant state with round tumor cell morphology.	[39]
H1975 lung cancer cells seeded above airway epithelial cells	A) - B) Lung microvascular ECs	A) Vertical MPS with two channels separated by a porous membrane B) BM coating C) Peristaltic pump D) ECL	• Tumor cells grow and spread more under static conditions than when exposed to breathing-like cues. • Mechanically stimulated tumor cells mimic the natural resistance of non-small cell lung cancer to treatment.	[40]

Abbreviations: MDCK, Madin-Darby Canine Kidney; ECM, extracellular matrix; MMP, matrix metalloproteinase; NFs, normal fibroblasts; Ecad, E-cadherin; BM, basement membrane; MVN, microvascular network; ECL, endothelial cell lining; ECs, endothelial cells; HUVECs, human umbilical vein endothelial cells; HLECs, human lymphatic endothelial cells; CTCs, circulating tumor cells; EMT, epithelial-mesenchymal transition; EVs, extracellular vesicles; EndMT, endothelial-mesenchymal transition; hBM-MSCs, bone marrow-derived mesenchymal stem cells; MSCs, mesenchymal stem cells; HAP, hydroxyapatite; GelMA, gelatin methacrylate

### Modelling tumor cell circulation

The circulatory phase of metastasis is a critical bottleneck for tumor progression [12, 23]. However, the chances of successful dissemination increase significantly when tumor cells enter the circulation as CTC clusters rather than as individual cells [124]. These clusters, composed of tumor cells and sometimes stromal or immune cells from their primary microenvironment [7, 44] are recognized as markers of advanced cancer progression, with their size and abundance strongly correlating with metastasis development [43]. CTC clusters exhibit a survival advantage in circulation, where they encounter new barriers related to innate immunity, oxidative stress, and tensions from circulatory shear stress [7]. Their resilience is attributed not only to physical integrity but also to increased drag force, which slows their flow velocity [43]. Capillaries with varying diameters and constrictions impose additional mechanical constraints, deforming CTCs and causing eventual arrest [124]. MPS models have proven highly useful for evaluating the impact of vascular microenvironmental factors on CTCs survival. These systems provide controlled flow conditions to perfuse single CTCs or clusters through channels with defined geometries [37, 62, 128, 141] enabling detailed analysis based on their physical and biological properties [139, 142].

To evaluate the combined effects of shear stress and physical constraints on CTCs, a MPS was designed with channels of varying geometries and evenly spaced microconstrictions, through which individual tumor cells were perfused [37]. The results revealed that perfusion and constriction-induced CTC deformation led to higher DNA damage and repair activity in SK-BR-3 breast cancer cells compared to MDA-231 cells, in which these mechanisms were constitutively active. This effect was accompanied by an upregulation of EMT-related genes in SK-BR-3 cells, along with an increase in their nuclear size. Nuclear deformation is critical for CTCs to navigate narrow constrictions [127] as highlighted in a study using a MPS consisting of a microconstriction array, through which CTCs were circulated under pressure-regulated conditions [128]. In this series of experiments, preferential deformation of the cytoplasm was observed at low pressures, whereas at tighter constrictions and higher pressures, cell deformation became dependent on nuclear stiffness. These findings also explained the greater deformability of MDA-231 cells, compared to MCF-7 cells, due to their smaller nuclear size, and the reduction in nuclear size and stiffness in the MCF-7 cells that successfully translocated.

CTCs represent a heterogeneous population of tumor cells with diverse phenotypes, transitioning between hybrid epithelial/mesenchymal or partial EMT states. This transition involves the integration of epithelial cell-cell adhesion and mesenchymal motility properties, which together turn these cells into highly potent metastatic effectors [124]. Similar to individual CTCs, CTC clusters display remarkable deformability, allowing them to pass through microfluidic channels with physiological diameters and perfusion pressures in a single file [62]. In a study, clusters isolated from blood samples of patients with malignant melanoma, as well as MDA-231 clusters, were shown to transit both endothelial cell-coated and uncoated capillary-like microchannels, recovering their original organization upon exit. Interestingly, treatment with paclitaxel (PTX) and focal adhesion kinase (FAK) inhibitors disrupted the integrity of these clusters, breaking them into smaller aggregates during transit [62].

To further investigate how the balance between mesenchymal and epithelial features influences CTC cluster integrity, Green and colleagues conducted a series of experiments using MDA-231 and MCF-10ADCIS clusters, both pure and genetically modified to express intermediate EMT markers (Ecad/Twist upregulation) [139]. Using the combined Pillar-X device, which integrates CTC profiling by physical properties and epithelial cell adhesion molecule (EpCAM) expression via magnetic nanoparticles, the study revealed that epithelial junction components are essential for cluster stability, while partial mesenchymal traits contribute to cluster survival by improving its deformability (Fig. 4B). Recent research underscores the role of EMT phenotype balance in metastasis treatment, revealing that the proportion of mesenchymal-predominant cells in CTC clusters increases as cancer develops resistance to therapy but sharply declines when an effective new treatment is introduced [11, 124].

### Modelling tumor cell extravasation

During systemic circulation, signals from distant organs and the unique characteristics of their vasculature dictate the site and mechanism of CTC arrest [19, 31]. This initial step, critical for extravasation, is influenced by physical factors such as vessel diameter [37, 128] and shear stress [20, 38, 114]along with specific interactions between CTC surface proteins and endothelial cells [41, 132, 143]. Circulatory patterns further determine the capillary beds encountered by CTCs, where they may become passively trapped in narrow capillaries or bifurcations, or actively adhere to vessel walls under optimal flow conditions [19, 140].

Regarding the adhesion mechanism, CTCs are more likely to stabilize and interact with endothelial cells in regions of low fluidic flow [7]. A study by Kamm’s team investigated this by examining the effects of varying flow velocities and pressures, simulating luminal and trans-endothelial flows, on the extravasation potential of MDA-231 cells using a horizontal MPS [114]. Luminal flow across the MVN in the central chamber was generated by creating a pressure differential between perfused lateral media channels using a pressure-regulated flow controller. Trans-endothelial flow was further induced by increasing the pressure to drive fluid across endothelial junctions, effectively replicating IFF and lymphatic drainage. These findings revealed that luminal flow significantly increased extravasation efficiency (approximately 3-fold compared to static controls) by triggering EMT activation in tumor cells and weakening endothelial junctions. Although trans-endothelial flow did not show a significant additive effect, it contributed to accelerating the rate of extravasation and subsequent cell migration into the matrix.

In addition, the glycocalyx (GCX) plays a crucial role in mediating the interaction between tumor cells and the endothelium. Composed of glycoproteins and proteoglycans, it forms a dense, charged, and hydrated barrier that helps maintain endothelial integrity and prevents the adhesion of tumor cells [38]. On tumor cells, however, the GCX may enhance adhesion, thereby promoting extravasation [144]. Disruptive events such as inflammation or high shear stress can damage the endothelial GCX, exposing it to degradation by extracellular proteases [140]. A previous study by Kamm’s group examined the role of endothelial GCX in tumor cell adhesion using a horizontal MPS [114] (Fig. 4C) perfused at a physiological flow rate [38]. They observed that CTCs deposit hyaluronic acid on exposed endothelial cells, which facilitates the adhesion of subsequent CTCs via the CD44 receptor. Furthermore, a positive correlation was identified between the expression of CD44 variant isoforms and cell malignancy. Beyond the GCX, coagulation factors such as fibrinogen can also enhance tumor cell adhesion. A separate investigation on a similar MPS [114] revealed that fibrinogen forms bridges between intercellular adhesion molecule-1 (ICAM-1) receptors on both endothelial and tumor cells, thereby increasing tumor cell adhesion and extravasation [143]. This effect was particularly pronounced in MDA-231 cells, which express higher levels of ICAM-1 compared to SKBR3 cells. Other procoagulants, including thrombin and fibrin, exibited the same effects, although to a lesser extent than fibrinogen.

Once arrested, CTCs can either extravasate through TEM or initiate intravascular growth, forming an embolus that could eventually rupture the vessel [7]. This unusual clustering behavior was observed in a study of breast cancer extravasation from a lymphatic vessel construct [75]. In this setup, a horizontal MPS with a matrix compartment between the lymphatic and vascular channels was used to examine the interactions between these systems and mechanisms of lymphatic extravasation. The circulating SK-BR-3 cells arrested and adhered to the sidewall of the lymphatic channel and extravasated into the central chamber upon IL-6 stimulation. Simultaneously, VEGF released by HLECs induced angiogenic growth from the vascular channel.

Transendothelial migration can occur either via paracellular routes, where cells pass through endothelial junctions, or via transcellular routes, where they migrate directly through endothelial cells. The transcellular route, however, is less common in vivo [19, 31]. In the paracellular pathway, tumor cells adhere to the endothelium and extend invadopodia to penetrate between endothelial cells and beyond the BM, facilitating their passage. Invadopodia are actin-rich protrusions whose function is closely linked to their interaction with focal adhesions, which anchor them to the extracellular matrix through integrin receptors [46]. FAK1 plays a critical role in this process by recruiting proteins like Talin 1, whose overexpression correlates with poor cancer prognosis. In a recent study by Moretti’s group, the roles of FAK and Talin-1 in extravasation were examined using MDA-231 and fibrosarcoma HT1080 cells, both of which commonly exhibit alterations in FAK expression [57]. The researchers utilized a three-chamber horizontal MPS model, with lung fibroblasts on the sides of the tumor chamber to mimic distant organ signaling. Their findings revealed that silencing Talin 1 significantly impaired tumor cell adhesion to the vasculature, while FAK inhibition had an even greater impact, notably reducing the tumor cells’ capacity for TEM.

Immune cells also play critical roles in extravasation, contributing to tumor cell adhesion [116] endothelial rupture [58] and TEM [145] among other processes. Using a high-throughput horizontal MPS design with 8 chambers, all filled from a single inlet and flanked by channels, Chen and colleagues demonstrated that neutrophils with an inflammatory phenotype interacted with circulating tumor cells and formed heterotypic aggregates [116]. These aggregates not only slowed the circulation velocity of CTCs but also increased their arrest efficacy through physical trapping and neutrophil-mediated endothelial adhesion. Moreover, the aggregated neutrophils secreted higher levels of IL-8, which disrupted nearby endothelial junctions, facilitating tumor cell extravasation. Neutrophil-platelet interaction can further potentiate MDA-231 extravasation [145] as demonstrated by Moretti’s group using the previously described horizontal MPS design [57]. In this study, treatment with the antiplatelet drug eptifibatide, an integrin β3 antagonist, effectively reduced platelet aggregation, downregulated EMT marker expression, and impaired tumor-endothelial adhesion.

In addition to neutrophils, circulating monocytes have garnered significant attention for their role in promoting tumor cell extravasation through endothelial opening, a process inherent to their function as immune surveillance agents [125]. In a research using a previously described three-chamber MPS, perfused monocytes were found to enhance the extravasation of MDA-231 through MMP9 secretion, which weakened endothelial junctions, facilitating their passage through TEM [58]. Once in the secondary stroma, monocytes differentiated into macrophages, and their movement created microtracks in the matrix, further promoting tumor invasion. Conversely, Boussommier-Calleja et al. observed a reduction in tumor cells extravasation in co-culture with monocytes and no significant changes in endothelial permeability [146]. This discrepancy likely stems from variations in experimental conditions, such as differences in endothelial cell types (HUVECs vs. human dermal microvascular endothelial cells) and the inclusion of NFs in the second study, which may influence the monocyte behavior.

Despite the unique advantages of vascular MPS models in replicating metastasis—offering a level of control and precision largely absent in animal models due to their limited monitoring capacities, lack of a complete immune system, and dificult-to-control physiological variables—several limitations persist. One of the main challenges lies in replicating realistic vascular perfusion and shear stress profiles. While the vasculature formed within these platforms can be perfused, maintaining stable, uniform, and biologically relevant flow conditions remains technically demanding and often inconsistent across models [129, 147, 148]. Another significant limitation is the simplified representation of the vascular niche, which frequently lacks integrated stromal or immune components—critical elements for capturing realistic tumor–vasculature interactions [130]. In addition, the absence of standardized benchmarks for validating vascular functionality directly contributes to poor reproducibility across laboratories, hindering both the comparability and translational value of these models.

Beyond these general challenges, there are also notable gaps in modeling specific aspects of the metastatic process. For instance, despite the well-established role of lymphatic vessels in metastasis in vivo, very few studies have examined tumor–lymphatic interactions, tumor cell migration across lymphatic endothelium, or the crosstalk between lymphatic and blood vessels in the context of metastasis [75, 149]. This limited modelling of lymphatic vessels in vitro largely reflects the technical difficulties of replicating lymphatic physiology—such as the structural and functional differences of lymphatic endothelium and the requirement for multiple distinct flow regimes [150].

A further underexplored area is the circulatory phase of metastasis, not only due to the difficulties in modelling it, but also because of the limited understanding of CTCs. One of the main reasons for this is the transient nature of CTCs, which typically survive only for hours or days—making them difficult to track in vivo and study in detail [19]. Nonetheless, in vitro research of this stage using MPS offers promising opportunities to advance CTC characterization. Which could ultimately improve their isolation and enrichment, with potential implications for diagnostic methods based on liquid biopsy [142].

On the other hand, although this section primarily focuses on the vascular aspects of extravasation, it cannot be fully understood without considering the influence of the secondary organ. For this reason, in the following section, these processes are revisited from a broader perspective, integrating the journey of CTCs and recreating their specific interaction with the distant organ’s endothelium.

## Microphysiological systems for studying metastatic colonization

Once CTCs reach a distant organ, the microenvironment they encounter becomes critical for supporting their survival and promoting metastatic outgrowth [7, 19, 39, 42] underscoring the concept of organotropism, the phenomenon whereby cancers preferentially metastasize to and colonize organs with a compatible microenvironment [41, 151]. This section examines the interaction between CTCs and distant organs during the final stages of metastasis, with particular emphasis on MPS that replicate the biological and biophysical properties of these tissues.

Beyond single-organ models, multi-organ MPS with interconnected chambers provide a comprehensive approach to investigate organ-specific metastatic tendencies across different cancer types [36, 152, 153]. For instance, one such study employed a multi-chamber MPS with a central compartment housing colorectal spheroids connected to parallel downstream liver, lung, and endothelial compartments. In a system with fixed flow rate and colonization distance, circulating HCT116 colon cells selectively adhered and proliferated first within lung constructs (A549 cells), followed by liver constructs (HepG2 cells), thereby successfully replicating the organ-specific metastatic patterns observed in colorectal cancer patients [36].

The host microenvironment, known as the pre-metastatic niche (PMN), is an active agent in metastasis, evolving in parallel with the tumor [44] and often becoming primed even before metastatic tumor cells arrive [13]. This priming is driven by secretory factors and extracellular vesicles (EVs) released by primary tumors into circulation, initiating processes such as vascular opening, ECM remodeling, stromal modulation, immunosuppression, and angiogenesis [20, 39, 154–156]which together prepare receptive organs for extravasation and colonization [31, 42]. A study on a vertical MPS demonstrated organotropism by showing that EVs from MDA-231 cells predominantly accumulated in liver tissues compared to kidney tissues, consistent with in vivo behavior [21]. This preference was linked to higher CXCL12 expression in liver tissues, which the MDA-231 EVs targeted through overexpressed receptors. This mechanism also explained the stronger liver tropism of MDA-231 EVs compared to those from less aggressive cell lines like MCF-7. These results were obtained using a two-compartment MPS that incorporated precision-cut liver or kidney tissue slices on a semipermeable membrane, with EVs perfused through endothelialized channels from below.

Junyoung et al. demonstrated the role of breast cancer-derived EVs in priming liver sinusoidal endothelial cells (LSECs) to promote CTC adhesion and vascular disruption for extravasation [20]. EVs from MDA-231 cells increased CTC adhesion threefold by upregulating fibronectin on LSECs through TGF-β1 signaling, a mechanism that was found to correlate with elevated TGF-β1 levels in EVs from breast cancer patients with liver metastases. This effect was highly specific, as EVs from other metastatic cell lines or replacing LSECs with HUVECs showed minimal adhesion increases. In addition, TGF-β1 in MDA-231 EVs induced fibroblast activation and hepatic differentiation, contributing to the formation of a liver-specific breast cancer PMN. The investigation of these interactions was enabled by a vertical MPS featuring LSECs in the upper channel and hepatocytes with liver fibroblasts in the lower chamber, mimicking the space of Disse. EVs were perfused through the endothelial space, followed by CTCs, to evaluate changes in adhesion caused by EVs priming. MDA-231 EVs were also found to promote vascular opening by inducing endothelial-mesenchymal transition (EndMT) in LSECs, a process by which endothelial cells transform into activated fibroblasts, compromising vascular integrity. Notably, melanoma-derived EVs have been found to trigger a similar EndMT in HUVECs, with a synergistic effect observed when combined with IFF [157]. This was tested using a horizontal MPS in which flow was generated by a pressure difference between lateral channels. Follow-up work using the same MPS design reported that these fibroblasts transdifferentiated from endothelial cells further contributed to a pro-metastatic effect within the established PMN [158].

Among distant organs, the bone, liver, and lung are of particular interest in late-stage metastasis studies because their vascular physiology, consisting of small capillaries and permeable sinusoids, makes them common sites of colonization. One example is the study by Bersini et al., who modeled the bone microenvironment (BME) in a horizontal MPS by seeding osteodifferentiated human bone marrow-derived mesenchymal stem cells (hBM-MSCs) within a dense collagen I matrix contained in the central chamber [140]. Using this configuration, the authors found that BME signaling selectively enhanced the extravasation and invasion of perfused MDA-231 breast cancer cells, while reflecting the varying degrees of bone tropism of ovarian (OVCAR-3) and bladder (T24) cancer cells, according to their levels of extravasation. In a more recent investigation, models of bone and lung breast cancer metastasis were developed by seeding osteoblasts, hBM-MSCs, or lung fibroblasts embedded in collagen matrices around rod-patterned vascular channels on horizontal MPS [74]. These systems demonstrated that osteoblasts specifically promoted bone-tropic MDA-231 extravasation, while hBM-MSCs and lung fibroblasts stimulated the extravasation of both bone- and lung-tropic cells indiscriminately. This effect of hBM-MSCs was attributed to their non-osteodifferentiated state, contrasting with the specificity observed in the previous study. In all conditions, acellular matrices exhibited the lowest levels of extravasation, while metastasized MDA-231 cells showed greater extravasation compared to parental, non-metastasized cells.

Even if tumor cells succeed in seeding a distant organ, their survival and growth are not guaranteed, as the secondary niche usually inhibits metastatic progression [44]. Consequently, tumor cells may enter metastatic dormancy, a late adaptation phase characterized by slow proliferation, increased drug resistance, and immune evasion [7]. This latency state can persist indefinitely, allowing the accumulation of genetic and epigenetic alterations before reactivation and aggressive colonization of the organ [41]. Disruptive events, like tissue damage or tumor-related signals, can trigger dormancy reactivation, as reported by multiple studies on MPS showing how an initially tumor-suppressive host-organ microenvironment can shift to support reactivation under these conditions [39, 40, 159–161].

In bone, dormancy reactivation is typically initiated by inflammatory signals from osteoclast-mediated bone resorption, a process often overactivated by tumor-secreted factors [39, 162]. This dynamic was replicated in a horizontal MPS featuring distinct niches mimicking the bone metastasis vicious cycle [39]. The dormancy niche was recreated using 3D-printed hydroxyapatite (HAP) and photocrosslinkable gelatin methacrylate (GelMA) hydrogels seeded with MSCs to simulate the complex bone matrix, while the reactivation niche was represented using osteoclasts in a GelMA hydrogel (Fig. 4D). These niches were connected by endothelialized H-shaped channels, which mimicked the characteristic bone perivascular niche, and were seeded with lung carcinoma A549 cells. This setup allowed for the observation that while co-culture with MSCs maintained tumor cells in a dormant state, osteoclast-derived signaling triggered dormancy reactivation, as indicated by increased invadopodia formation.

On the other hand, emerging evidence suggests that physical activity may help mitigate or prevent cancer-induced bone damage and metastasis. A recent investigation conducted on a horizontal MPS supports this hypothesis. The device featured a BME channel containing MLO-Y4 osteocyte-like murine cells seeded on a collagen coating, connected by a matrix compartment to a luminal channel lined by HUVECs, with MDA-231 cells seeded on top [163]. Additionally, the system incorporated a custom pumping mechanism to apply oscillatory flow to the BME channel, simulating the shear stress caused by bone loading during exercise. The study demonstrated that mechanically activated osteocytes decreased the rate of extravasation and reduced the distance traveled by MDA-231 cells from the luminal channel, an effect not observed in experiments without osteocytes or flow stimulation.

The role of mechanical cues in tumor dormancy was also investigated by Ingber’s group. Using a vertical MPS design to replicate the lung microenvironment, the authors found that respiratory motion-mimicking stimuli suppressed NSCLC growth and invasiveness, suggesting that alveolar obstruction caused by tumor growth may trigger latency reactivation [40]. The MPS consisted of an endothelial channel at the bottom and a lung epithelial channel at the top, where the medium was removed after 3 days to establish an air-liquid interface for lung differentiation. To simulate respiration, the design incorporated two adjacent parallel microchannels on the sides, through which cyclic mechanical deformation was induced via a peristaltic pump. After two weeks of culture, NSCLC cells grown under static conditions formed large clusters and expanded into the vascular channel, whereas breathing-like motions reduced these effects by half. The platform also recapitulated NSCLC resistance to tyrosine kinase inhibitors, erlotinib and rociletinib, which was linked to mechanically induced epidermal growth factor receptor (EGFR) downregulation.

In relation to the other metastatic steps, replicating late-stage metastatic events presents additional difficulties, particularly considering the need for a multi-organ approach and the integration of vasculature. Current colonization models—in particular organoids, commonly used to assess the local adaptation of tumor cells and which typically lack vasculature, and animal models, which have very low efficiency in orthotopic colonization, offer limited capabilities [19, 32].

In this context, while emerging multi-organ platforms — primarily single MPS units with interconnected chambers [36, 152, 153] – show potential for studying cancer organotropism and inter-organ interactions, their overly simplistic design, particularly in terms of vascular integration, limits their ability to fully capture the complexity of metastatic colonization. In contrast, single-organ models replicate the secondary TME more accurately but miss the influence of the primary tumor. This compartmentalized approach, while informative, limits the integrated understanding of metastasis as a continuous process. The ideal scenario would involve comprehensive models that integrate the entire primary tumor-vasculature-secondary niche axis—whether in a single MPS or through fluidically interconnected platforms [164, 165]. This would merge the strengths of both approaches, enabling in-depth analysis of critical events like early PMN priming, tumor dormancy regulation, and phenotypic changes during tumor progression.

## Challenges and future perspectives

MPS have greatly advanced our understanding of metastasis by providing a focused approach to each step, enabling the integration of microenvironmental factors with precise experimental control and continuous high-resolution monitoring of tumor cell dynamics and treatment response. Moreover, their recent endorsement by the Food and Drug Administration (FDA)—alongside other human-based technologies such as organoids and computational models—for testing monoclonal antibody drugs, through the launch of a strategic roadmap (April 2025) aimed at progressively eliminating the requirement for animal testing, underscores their growing role in preclinical research [166, 167]. However, metastasis MPS models still face challenges in fully recapitulating tissue-specific conditions, and technical limitations continue to restrict their preclinical applicability and translational potential.

Repliting the pathophysiological context of the primary tumor and its surrounding TME without compromising experimental readout and reproducibility remains the central challenge in MPS-based metastasis modeling. Many existing MPS models lack the heterogeneous scaffolds, cellular diversity, and tissue maturity needed to emulate in vivo conditions, as they often rely on homogeneous tumor cell lines and commercial matrices. Although recent studies have begun incorporating native tissues—such as decellularized extracellular matrices [168] primary cells [20, 103] and organoids [35, 71, 169]—to better capture tumor histopathology and intertumoral heterogeneity, these approaches introduce additional technical challenges related to inter-patient variability, limited scalability, and complex handling, that currently limit their widespread use. These difficulties are further compounded by the methodological complexity involved in integrating relevant microenvironmental mechanical stimuli, such as laminar shear stress or cyclic strain, which often require specialized knowledge and infrastructure, thereby restricting their accessibility and standardization [147, 170].

These design complexities also translate into analytical limitations. Although 3D ECM-based systems offer greater biological relevance, they complicate downstream analysis due to poor cell recovery and extraction challenges, which hinder the application of essential molecular techniques such as flow cytometry, Western blotting, qPCR, single-cell RNA sequencing, and high-throughput omics. In addition, conventional non-destructive real-time monitoring approaches, while compatible with most MPS, often lack sufficient sensitivity and resolution when applied in these systems, limiting their ability to capture subtle dynamic changes or rare events within the TME. Taken together, these constraints reduce not only the analytical depth achievable in such platforms but also their compatibility with large-scale or longitudinal studies.

In response to these limitations, multidisciplinary and collaborative efforts are emerging to improve not only the biological fidelity and interpretability of MPS but also their standardization and scalability for preclinical research. On the analytical front, increasingly sophisticated real-time monitoring approaches are being developed, tailored to the unique features of MPS. These include integrated biosensors—such as optical and electrochemical sensors [171–173]—that enable non-invasive, continuous tracking of physiological, biochemical, and mechanical parameters such as oxygen, pH, or fluid flow rate. These tools, although still limited in their application to metastasis research, could be particularly valuable for detecting hypoxic tumor cores, mechanically constricted microvascular regions, or metabolic shifts driven by tumor-secreted factors in the PMN. Additionally, non-invasive techniques like surface-enhanced Raman spectroscopy (SERS), which can be integrated into MPS platforms via nanostructured substrates, enable highly sensitive molecular characterization and hold promise for near real-time analysis [174]. In parallel, digital modeling strategies are gaining traction, with machine learning and computational simulation being used for dynamic analysis and predictive modeling of tumor–TME interactions or drug responses [175–177].

On the other hand, to address standardization challenges, efforts are being made to reduce the fragmentation of the MPS landscape by promoting modularity and interoperability across platforms. This is being facilitated through adherence to ISO guidelines and the adoption of standard microwell plate form platforms [130]. Scalability is also advancing, driven by the development of parallelized and multiplexed MPS systems, automated cell seeding techniques, and closed-loop platforms that incorporate real-time environmental sensing and feedback control to maintain tissue homeostasis [130, 148]. Finally, 3D bioprinting is being explored as a strategy to increase the architectural and functional complexity of MPS in a format compatible with automated workflows, although its widespread adoption remains in its early stages [149, 178].

In conclusion, although MPS of functional organs are already incorporated into preclinical pipelines for evaluating drug toxicity, metabolism, and functionality [166], tumor-specific MPS models still face limited clinical validation and insufficient operational standardization for broader implementation [130, 179]. Metastasis models, in particular, present added challenges due to their greater biological complexity. Nevertheless, thanks to emerging technological innovations, the outlook is promising, especially considering their potential integration with organoids, particularly through vascularized multi-organ models [155, 169, 180] and with computational approaches, a combination that could enhance both their biological relevance and predictive value for clinical decision-making.

## Data Availability

No datasets were generated or analysed during the current study.
